# Integration and social determinants of health and wellbeing for people from refugee backgrounds resettled in a rural town in South Australia: a qualitative study

**DOI:** 10.1186/s12889-020-09724-z

**Published:** 2020-11-13

**Authors:** Anna Ziersch, Emily Miller, Melanie Baak, Lillian Mwanri

**Affiliations:** 1 Southgate Institute for Health, Society and Equity, Flinders University, GPO Box 2100, Adelaide, SA 5001 Australia; 2 College of Medicine and Public Health, Flinders University, GPO Box 2100, Adelaide, SA 5001 Australia; 3 School of Education, University of South Australia, St Bernards Rd, Magill, SA 5072 Australia

**Keywords:** regional, rural, social determinants of health, resettlement, refugee, health, employment, education, housing, integration, well-being

## Abstract

**Background:**

There has been a recent focus on resettlement of migrants and refugee in rural settings in Australia and elsewhere. Rural resettlement is seen as an opportunity to revitalise rural communities, to fill the needs of employers in these areas, and to provide a welcoming community within which new arrivals can integrate and settle. However, challenges to rural resettlement have been identified including difficulties securing employment, discrimination and social isolation. These challenges can affect resettlement outcomes including health and wellbeing, though relatively little research has examined these links. In this paper we explored experiences of people from refugee background settling in a rural Australian town, examining interconnections between social determinants of health (SDH) and integration.

**Methods:**

Face-to-face interviews were conducted with 44 participants from Southeast Asia and Africa in a rural setting in South Australia, covering experiences of resettlement and impacts on health and wellbeing. Participants were recruited through existing connections within the community and snowball sampling. Audio recorded data were transcribed verbatim and analysed using framework thematic analysis.

**Results:**

The study findings revealed a mixture of settlement experiences for participants across a range of elements of SDH and integration. A sense of safety and some elements of social connectedness and support were key enablers for integration and health and wellbeing, with main challenges including limitations in employment opportunities, mismatched education provision, experiences of discrimination and constrained access to services.

**Conclusions:**

Challenges experienced by refugees resettled in rural areas can affect integration, health and wellbeing and subsequent onward migration intentions. Attention to broader socioeconomic, cultural and environmental conditions, alongside tailored settlement support policies and practices for individual rural resettlement sites, is required to support integration and health and wellbeing.

**Supplementary Information:**

The online version contains supplementary material available at 10.1186/s12889-020-09724-z.

## Background

Globally, there are currently almost 80 million forcibly displaced people, including 26 million refugees and over 4 million asylum seekers [1]. Refugees and asylum seekers flee their countries because of persecution, war and/or violence and many are exposed to torture and trauma [2]. These pre migration factors and also post-migration factors including challenges settling in a new country like learning a new language, securing employment and making social connections, mean that refugee status is associated with poorer health outcomes, particularly mental health [3, 4]. There is a need to unpack the complex post-resettlement pathways contributing to this inequity. A consideration of integration and the social determinants of health (SDH) offers insights into this [5]. Research with a focus on SDH has proliferated in the last decade [6] and connections between aspects of integration and health are beginning to be highlighted [7]. However, experiences of SDH for people from migrant or refugee backgrounds are less well documented overall, particularly in non-metropolitan resettlement settings [8]. As rural resettlement priorities are increasingly a focus of governments, particularly in Australia, understanding the relationships between integration and SDH in these environments is key to successful resettlement outcomes [9–11]. In this paper we focus on experiences of people from refugee backgrounds settling in a rural Australian town and examine the interplay between integration and health and wellbeing in this context, in order to inform settlement policy and practice.

### Integration and the social determinants of health

SDH are the ‘conditions in which people are born, grow, work, live, and age...[which] are shaped by the distribution of money, power and resources at global, national and local levels’ [12]. SDH can be considered to be ‘the causes of the causes’ of health inequities [13]. Dahlgren and Whitehead [14] in their influential SDH model highlight relevant conditions at various levels that affect health - individual factors (e.g. age, sex), individual lifestyle factors (e.g. exercise, smoking), social and community networks, living and working conditions (e.g. education, work environment, housing) and general socioeconomic, cultural and environmental conditions. For people from refugee backgrounds in resettlement contexts, some of these conditions of life impact not only on health outcomes, but also the experience of settlement, with integration an integral part of that process.

Integration is a contested concept [15–20] but is understood here as a two-way process of adaptation that occurs between people arriving in a new place and the receiving communities [19, 21]. In countries of resettlement, facilitating successful integration can have multiple social, economic and health benefits for both refugees and receiving communities - likewise challenges to integration can have detrimental effects for all [15, 19, 21–27].

Ager and Strang’s [19, 21]‘s influential framework of integration includes ten indicators of integration across four domains. In the ‘Means and Markers’ domain, housing, education and employment, and also health, are seen as markers of, and also a means to, successful integration. The ‘Social Connection’ domain comprises social bonds, bridges and linkages. ‘Facilitators’ includes language and cultural knowledge, and safety and stability, and ‘Foundation’ deals with rights and citizenship. This model overlaps considerably with Dahlgren and Whitehead’s work on the determinants of health and the shared markers of SDH and integration include education, housing, and employment, with social connections and networks as key facilitators in both frameworks, and health an element of interest for both. Hence, the ways in which people from refugee backgrounds are able to settle in new places, through accessing appropriate services, jobs, schools, housing and community networks can ultimately affect their health outcomes [5]. Thus, there are key intersections between elements of integration and SDH that are important foci for researchers, service providers and policy makers working towards successful resettlement outcomes. While a SDH approach can offer important insights into how social factors beyond individual behaviours, such as those involved in integration, influence health [12, 13, 28], there has been relatively little literature that has explicitly linked refugee integration and the social determinants of health [5, 29, 30].

### Regional resettlement in Australia

Globally, displaced people most often continue life, if possible, in neighbouring regions [1]. Less than 1 % of refugees are resettled in countries of the global north, such as Australia [31]. The total number of resettlement places offered by Australia has fluctuated somewhat over time, with an annual total of 18,750 places for the 2018–2019 period [32], a figure which represents less than 10 % of net migration to Australia [33].

Historically, people from refugee backgrounds settling in Australia tended to move into cities where services are more available and accessible. In recent times the Australian government has prioritised rural settlement - termed regional settlement in the Australian vernacular - for both temporary migrants and permanent new residents [9, 34–36]. As a result, the proportion of people with refugee backgrounds in rural towns has increased significantly over the past decade [34, 35]. This formal rural settlement focus is relatively unique. Internationally, there have been dispersal programs, particularly in Europe in managing large influxes of asylum seekers [37–39]. However, these have been more to smaller cities rather than rural or regional areas, and in many cases have involved compulsory elements and punitive measures. In some countries, such as Canada, greater numbers of refugees have settled in rural areas through community sponsored refugee settlement [40], rather than a targeted government program.

The prioritisation of rural resettlement is pursued for a variety of reasons. In Australia it is viewed as a way to revitalise rural communities, to fill the needs of employers in these areas, and to provide a welcoming community within which people can integrate and settle [41, 42]. Rural resettlement policy in Australia has tended to settle people from similar cultural backgrounds in clusters in specific towns throughout the country [41, 43] – for example, the town of interest in this study has concentrations of people from Burma/Myanmar and the Democratic Republic of Congo.

The potential for rural resettlement policy priorities to benefit rural towns as a whole, and migrant or refugee individuals, families and communities is counterbalanced by the potential challenges presented in rural settings. Economic and employment factors are vital to successful settlement [44], and people from refugee backgrounds may show willingness to work in jobs that are less desirable, hence filling employment niches [43]. However, work satisfaction may be limited in these jobs, and social aspects of wellbeing and life satisfaction are also crucial [45], particularly as a major rationale for humanitarian resettlement is that people are supported to live a safe and connected life in their new community [9].

In Australia the humanitarian settlement scheme offers settlement services to people during the initial settlement phase. The support includes help with familiarizing with local services, free attendance at English language classes, and temporary housing. This support is only short term (at the time of data collection up to 6 to 12 months) and people are eventually expected to transition to less specialised services and to be successful in providing for themselves within the Australian systems. The ways in which towns are able to offer services and employment, as well as a welcoming community, have a strong impact on integration and SDH for people from refugee backgrounds [41], and hence on their ongoing health and wellbeing [46]. This is particularly important initially as focused settlement support services may be more limited in rural areas [47], and longitudinally as settlement services are replaced by expectations that people will access non-specialist services. Health and wellbeing in the initial stages of settlement can be quite different as time in a new home country goes on [48]. ‘Mainstream’ services may have little understanding of the unique experiences and skills of people from refugee backgrounds [49]. Overall settlement outcomes may differ depending on various factors including, for example, employment [50], gender [51], age [52], or housing [53].

Research investigating the broad resettlement experiences for people from refugee backgrounds in high income countries has proliferated in recent years [54], however less research has focused on the experiences of refugees in regional or rural areas, particularly regarding health in those environments [55], and there is very limited research outside Australia on the experiences of refugees in regional or rural areas. The research that has been conducted has indicated that successful settlement is dependent on a number of factors [11, 47, 56, 57] such as attitudes of the host community [41]; rural employment opportunities [58]; and size and connections of the incoming community [59], and highlights the importance of local contexts for refugee resettlement [15, 60].

Here we report on a study with participants from refugee backgrounds who had settled in a rural town in South Australia, which aimed to examine how integration factors and SDH were experienced by new arrivals. We detail the ways in which key SDH including social connections, employment, education, housing and access to health services influence the settlement and integration of recent arrivals from refugee backgrounds. We do so in order to develop understanding of the ways that integration affects SDH and vice-versa, and ultimately to use this knowledge to identify practices that contribute to positive resettlement outcomes including health and wellbeing for people from refugee backgrounds and receiving communities.

## Methods

### Study setting

This study was conducted in a rural town of less than 30,000 people in South Australia during 2016. We will not name this place for confidentiality reasons but shall henceforth refer to it as The Town. Most residents of The Town have Anglo-Australian heritage and nearly 90% speak English at home [61]. On average in Australia approximately 70% of households speak only English at home, indicating that the level of cultural and linguistic diversity in The Town is more limited than in other areas of Australia, particularly urban areas [61]. Levels of education, median weekly income and occupational level in The Town are below the South Australian and Australian average [61].

The Town has been a focus for regional resettlement under the Australian federal government’s Refugee and Special Humanitarian Program since 2007, with a settlement program for refugees from Burma/Myanmar commencing then, and a more recent program of resettlement for refugees from Africa (largely from the Democratic Republic of Congo). In the 2016 Australian Census, the top language spoken in The Town, other than English, was Karen indicating the significance of the this community in the town [61].

### Sampling

The researchers recruited participants through existing connections with two community leaders, community networks and snowball sampling. This method of recruitment was a strength of the project in that it did not rely upon service providers to link researchers to participants, hence participants need not be accessing services to be invited to participate. The researchers explained the aim and scope of the research to a key community leader from each community who then invited other community members to participate. Individuals then referred the researchers on to friends or family who were eligible to be invited to participate by phone or face-to-face. No participants directly approached refused.

### Participant characteristics

In total the study included 44 participants aged between 18 and 68 years of age, with 22 women and 22 men (Table 1). They broadly had backgrounds in Burma/Myanmar and regions in and around the Democratic Republic of Congo (DRC). For simplicity’s sake, we use the term ‘Southeast Asia’ and ‘Africa’ to show characteristics of each group, however we would like to acknowledge that the individuals had a diversity of experience and heritage and these terms do not accurately represent this diversity. Almost all had been in Australia for less than 10 years.

**Table 1:** Participant characteristics

Age Range (***N***)				
	< 25 years	26–50 years	51–68 years	Unspecified
Southeast Asia	3	13	5	
Africa	1	14	4	4 (all older than 25)
**Gender (N)**				
	**Male**	**Female**		
Southeast Asia	11	10		
Africa	11	12		
**Length of time in Australia (*N*)**				
	**< 5 years**	**6–10 years**	**> 10 years**	**Unspecified**
Southeast Asia	11	10		
Africa	12	8	1	2

### Interviews

Interviews were conducted in 2016 by a core research team with support from paid interpreters where relevant. All of the team conducting interviews (AZ, LM and another researcher) were female and were university academics and experienced researchers. Two of the team were from non-migrant backgrounds, with the third originally from Africa. All but two of the participants were not previously known to the interviewers and in the cases of those known someone from the team who did not know the participant conducted the interview. Participants were given a choice of participating using English or with support from interpreters. Interpreters were known to participants and trust was built quickly with researchers over a short time, enabling effective data collection via frank conversations. Interviews were conducted in participant homes and in one case a local hotel room. Incidental people such as family members in the home were verbally informed about the research. Interviews were semi-structured and covered a broad range of experiences of life in The Town including housing, employment, social networks and support, and settlement support (see Supplementary file). Interviews ceased when data saturation was reached. Interviews ranged from 18 to 63 min with an average of 35 min and field notes were taken during and after the interview.

### Analysis

Individual interviews were recorded and transcribed, and drawing on grounded theory [62] analysed inductively using framework analysis which included five stages of analysis – familiarization with the data, development of a coding framework, applying the coding framework, charting the key themes, and mapping and interpretation [63]. Data analysis utilized NVivo Version 11 to assist in the coding of the interview material. Two of the team coded the transcripts and the four researchers worked together to collectively identify the themes outlined below and to interpret the data. The findings were included in a brief summary report which was circulated to the participants through community networks. We include excepts from the interviews below, with participants identified by pseudonym (which includes more than one name for those from Burma/Myanmar to reflect naming conventions), continent of origin and gender.

### Ethical approval

This study was approved by the Flinders University Social and Behavioural Ethics Committee (project 7101). The researchers ensured that informed consent was obtained prior to commencing the interviews, and interpreters assisted at this stage if required. Participants were given assurances of confidentiality and anonymity and the right to withdraw from the study at any time, and the lack of association of the researchers with services assisted in reassuring potential participants that their access to services was not affected by their participation or otherwise in the research. Interviews largely occurred within the participants homes to ensure that they felt comfortable and in a more empowered position. As noted, interpreters trusted by the participants were used.

## Results

Overall participants were very appreciative of being resettled in Australia. Almost all participants moved straight to The Town upon arrival in Australia, with a few exceptions of people moving to The Town by choice after first living in a capital city. Most were resettled in The Town by the Australian government resettlement program because of existing family connections. Their experiences in The Town reflected a number of health promoting features such as a sense of safety and security, a quiet atmosphere, a good place to raise children, community support and affordability. However, participants also described difficulties securing employment and a churn in education and employment, barriers to community engagement, experiences of racism, a lack of cultural resources, limitations in settlement support, and difficulties in service access due to travel requirements. Participants described a number of specific health concerns such as high blood pressure, heart conditions or diabetes, as well as more generalized issues relating to mental health and wellbeing, particularly stress and sadness. Key links were suggested by participants particularly in relation to issues with employment, discrimination and access to services and impacts on mental health.

In this section, we consider participant’s experiences through the lenses of SDH and Ager and Strang’s 2008 integration framework. Below we look at key areas of rights and citizenship, safety and belonging, social networks, employment, education, housing and access to services. We then discuss how negative experiences of some of these key elements of integration resulted in secondary migration usually to metropolitan areas for some people. We note the direct intersections between key areas of integration and SDH frameworks including safety and belonging, social networks and support, employment, education, housing and access to services and resources.

### Rights and citizenship

One of the foundational elements of Ager and Strang’s framework of integration is the importance of rights and citizenship [19]. These rights affect how people can access key SDH such as health services, housing, employment and education. In Australia there are a range of legal pathways that people from refugee backgrounds navigate to attain permanent residency and, for some, citizenship. These pathways critically impact experiences of resettlement. All of the participants in this study were either permanent Australian residents or citizens with refugee backgrounds, hence they legally had a reasonable level of access and rights. We acknowledge the current fraught political and experiential landscape in Australia for people seeking asylum, but these issues are beyond the scope of this work.

### Safety and belonging

A sense of safety and belonging is crucial to positive resettlement experiences, particularly for people who have had a refugee journey that intrinsically involves disrupted or interrupted feelings of safety. A sense of feeling safe was paramount to participants. Participants felt that The Town was a good place to bring up children, away from the dangers of war and persecution, as highlighted here by Celeste:

*Celeste: The most important thing about settlement, I just like the way they receive people here and it’s just a safe place to be.*

*Interviewer: Do you think that people feel that when they come here?*

*Celeste: Yeah, compared to where you are coming from, if it’s a place where there is war, even when you go to another country you just feel that relieved. (Celeste, Africa, female).*

Anton reiterated this sense of safety:

*[The Town] is a good place because it’s a place where you can live with your kids in a good condition than big cities […]. Even friends from there [other cities], they always tell us that ‘the place where you live, it’s a very, very good place to keep safe your kids for their small age’. (Anton, Africa, male).*

However, these feelings of safety were sometimes challenged by experiences of discrimination and racism in the Town. Maung Arkar described how he felt welcomed initially, but as time went on he and his family experienced racism. This made them feel unwelcome and unsafe.

*He said that when he came first in Australia he found that the people are very friendly and nice but -- and if we [live here a bit longer] then they are like -- they don’t care about much anymore […] sometimes he feels like not safe and […] feeling people don’t like me anymore so […] we want to go back to our country. (Maung Arkar (*via *interpreter), Southeast Asia, male).*

Other participants described experiencing discrimination in day-to-day life (“when you go in a shop, some shops …. You just enter in there and someone will just look at you as if they haven’t seen you” (Celeste, Africa, female), and in relation to structural interactions with employers or when trying to access services or resources such as housing (see below). These experiences undermined a sense of belonging and safety and integration.

### Social networks and support

A sense of safety and belonging were supported by the connections that participants developed and maintained. These were strongly represented by relationships within cultural and faith communities. Longer term residents of The Town offered some support for newcomers, and in turn these newcomers offered support for others who arrived in later years. For example, Etienne described difficulties in setting up his house including having a broken stove fixed, during which time other African community members, who he did not previously know, provided food and assisted him in having the repairs undertaken.

*He was really happy to find some people that did care about him when he had that matter and he was really, really affected and it was really impressed according to him because in time of having that difficult time people came to him and then helped him. (Etienne (*via *interpreter), Africa, male):*

Connections through churches, both with their cultural or ethnic communities as well as the broader community, were highlighted by many participants as offering spiritual support and friendship, but also practical supports such as driving, helping people find services or resources, or navigating other settlement issues as highlighted above in Etienne’s case:

*Those people, I mean they’re church people. They really, really cared about me since I have got that problem* [family health issues and travel to the city for specialist services] *until I’ve got a job* (Emmanuel, Africa, Male).

*From the church? Yeah, they do, like some of the Australian people they help migrant to get driving licence as well. Like when they have time they just, you know drive for them* (Zeyar Htet, Southeast Asia, male).

Anton also talked about a supportive relationship with his neighbours:

*Even neighbours, some of them are good, because I remember my first place where I moved, my first house, I had a neighbour; that neighbour was just like a father, not even like a friend. He was a good man and he has a good wife. We were living in a union like a family (Anton, Africa, male).*

However, English language skills were identified as a barrier to connecting to the broader community. For example, Zin Maung and Marie, both speaking through an interpreter said:

*Because he can’t communicate because he doesn’t speak English, so it’s a big issue here, so he can’t involve with the community, socialise.* (Zin Maung (via interpreter), Southeast Asia, male).

*The only barrier that I face is the language. Because I can’t speak very well English it’s very hard to interact with Australian people.* (Marie (via interpreter), Africa, female).

In this way community connections can be a key support for health and wellbeing, both in the initial phase of settlement and over time, with participants outlining the various ways that their relationships with people, particularly from their own communities, helped to connect them to appropriate services and successfully navigate Australian systems. However, participants indicated various experiences of discrimination or racism from the wider community and language issues made building connections more difficult.

### Employment

Prior to settling in Australia, participants had quite different experiences of employment. The Southeast Asian participants had predominantly worked in subsistence farming or as labourers, with the exception of two who had worked in the medical field. In contrast, while some of the African participants had been subsistence farmers, most had backgrounds in cities, employed in white collar work such as running a business (local and international), teaching and as medical professionals.

In Australia at the time of the study, the majority of participants including those who had been in Australia for some years were unemployed (60%), or in part-time, seasonal or casual work. No participants had secured full time ongoing work. Participants who were in paid employment were working in a meat processing plant, forestry, farming, aged care, cleaning, or in a cultural liaison role such as interpreting or providing support in education settings.

Aveline’s comments reflected many people’s experiences of difficulty finding work with limited opportunities available:

*To get settled here, it wasn’t really hard but the main thing that still struggle exist up to now is how to get a job. It’s very hard to get a job around here […] because I’ve been trying several times -- just imagine, I’ve been here for about six years but I’ve tried. The only opportunity we can get is those seasonal jobs, planting trees, something like that. (Aveline (*via *interpreter), Africa, female).*

Difficulties in having overseas qualifications recognised and a lack of Australian employment experience was highlighted by participants as a barrier to securing employment, as Henri says:

*Tried to get my qualifications recognised. It was too hard, expensive and then too much formalities. I didn’t have finances to afford that. […] When it comes to skilled things I think I’ve got all the capability to deliver but since here they don’t consider even our African experience, now to get the experience, if when you go to somewhere to ask even to be a volunteer so that at least you have something to write on your CV, no. (Henri, Africa, male).*

Henri’s difficulties in getting qualifications recognised meant that he was expected to seek work in other casual, short-term labouring roles which were a mismatch for his skillset. Age and (dis) ability were raised as potential barriers to employment for a number of participants. Some people had health issues that precluded them from certain types of work. Others, as Henri further explains, found that their older age meant that employers were not interested in hiring them.

*Like other job, like pruning fruit or what, it’s not something which is permanent, it’s maybe one month or two month people goes there, but sometimes I look at my age and …. I [can’t] go there … because I’m ageing. (Henri, Africa, male).*

Interrupted education and English language barriers were also highlighted, particularly in relation to the Southeast Asian community, many of whom had spent extended periods in refugee camps. For example, Zeyar Htet noted:

*So people live in the refugee camp their whole life, like they settle there for 20 years I think, 20 or 30 years, but those people, they don’t have education, you see? They can’t read ABC so those people are -- so they don’t have education so when they come here they settle here in [The Town] but they are over like 30 or 40 years of age you see and that’s the problem. How are they going to do it, get employment, because they don’t speak English? (Zeyar Htet, Southeast Asia, male).*

Discrimination was also highlighted, particularly by participants from Africa as limiting access to employment options. For example, Bondeko said that there were many available jobs in The Town particularly in aged care and that a number of members of the African community had been undertaking training courses in the area but had not been able to secure work:

*Many people are doing that course but some, they don’t have the job. I don’t know what is going on. I think the people for here, I think they don’t like African people because when go and apply for job it is not easy to get job. (Bondeko, Africa, male).*

Sabine likewise stated:

*I would say that it’s very hard to get a job. Especially when you are African it’s very hard to get a job. People do not give that opportunity to African to get a job* (Sabine (via interpreter), Africa, female).

Experiences of racism were also reported within the workplace. For example, Tatiana said:

*Maybe people here in [The Town], they don’t used yet to Africans I think; I don’t know. Even in aged care where I am working, some residents, they don’t like black skin and they will tell you straightforward … ‘Don’t get your black skin on me’ (Tatiana, Africa, female).*

Participants who had connections within the community built on these to find paid employment. Connections were variously through community members such as family and friends, through church communities, or connection to longer term residents of the town such as teachers. Reputations of individuals were spoken of as being perceived as a reflection of the broader ethnic communities, for example if a few people were employed in one organization this could lead to others with similar backgrounds being employed, as described by Zeyar Htet.

*Especially the aged care it’s easy because … there are two or three member […] from our background. You know, they work there and then they work very hard and that’s why their boss is very happy with their work. So then I went there, I applied for the work placement and then after ten days later I asked them if I could work here after I finished my certificate and they say yes. (Zeyar Htet, Southeast Asia, male).*

Volunteering was a common pathway to paid employment for participants. Although this ended in paid work for some people and was a successful way for people to gain experience and connect with employers, there was potential for people to be taken advantage of when working for free in roles that would usually be paid. Several participants described long periods of unpaid work, such as Myint Zaw Zaw who reported volunteering at an aged care facility for three months – “helping, cooking for the residents and also cleaning, washing and pushing the residents on the weekend around.”

The stress of not finding paid work and the financial implications were particularly mentioned by participants from African backgrounds, including Anton and Aveline:

*It was very, very sad. Very sad. You know, living here without doing anything, like just miserable …*. I *cannot be happy because it’s hard, very, very hard to live without job because there is many things which need money and there is no way you can get money without job* (Anton, Africa, male).

*When there’s no money you can’t respond to those kinds of needs … ..Like in Africa when you don’t have a job you don’t have consideration in the community so it’s not good … ..It’s very hard. You can feel like you are sick. You can feel like you are not in your good mood because you have to think about ‘I should be doing something. I should be doing something’ and that really, really make you stressful. (Aveline (*via *interpreter), Africa, female).*

Zeyar Htet also referred to members of the Southeast Asian community becoming ‘depressed’ because of difficulties finding work. In this way challenges relating to employment were crucial to participants’ health and wellbeing. Feelings of stress or depression were directly mentioned as being linked to unemployment, to seeking employment and to experiences in the workplace. Barriers to integration also stemmed from these employment related issues, as participants felt excluded and discriminated against.

### Education

Many participants with Southeast Asian heritage had relatively low levels of education prior to moving to Australia. In Australia, humanitarian settlement services provide funding for English classes during the initial phase of settlement [64]. This opportunity was available to all participants who arrived via the humanitarian program, and many Southeast Asian background participants mentioned their engagement with these classes. There were different levels of engagement with the classes. Some participants highlighted the importance for them to learn the Australian accent. In some cases, the English classes offered were inadequate because the opportunities to practice with native English speakers was limited:

*English is not easy language, right, and also … some Aussie people, they speak like Aussie accent, so sometimes like when -- he know the meaning but when he speaks it sounds different so to him, he thought like a bit difficult for him to understand because sometimes the teacher use Aussie language -- Aussie accent.[…]. And also he said that maybe just put him in line with English people so he can practice more his English, but […] the teacher put him and like all [Southeast Asian] people together […] most of the time they speak like their language.* (*Maung Arkar (*via *interpreter), Southeast Asia, male).*

Participants with African backgrounds had a range of education experiences prior to moving to Australia, but most had completed high school (or equivalent) and many had university level education. Participants with overseas qualifications expressed frustration at not having them recognised in Australia. Not only were overseas qualifications not recognised, but participants were encouraged to enroll in Australian study that was far below their capacity. For example, Celeste described a background in IT before coming to Australia:

*I was thinking maybe when we come here … .people would just go ‘continue from where we stopped’ but when we came here they told us we had to go and do English and [numeracy] at TAFE so going backwards because they said ‘unfortunately our papers are not accepted in Australia’ and...Then you go in a class where you are able to write, speak and understand what they’re talking about; it’s more like you’re going to a kindergarten … .. It was more like I was a kid being taught ABCD and all that. I wanted to do something more. (Celeste, Africa, female).*

Although engagement in education in Australia was high, this did not always guarantee employment. Many participants spoke of multiple qualifications gained both overseas and since settling in Australia. For example, Gabi, a registered nurse in her home country recounted undertaking the English program at TAFE soon after arrival, then a child services qualification, and then an aged care qualification as her nursing qualification was not recognised. She had still not been able to secure employment.

Many participants described persistence in trying to gain the right kinds of qualifications to secure employment. People expressed frustration about the lack of clear pathways from education to employment, as reflected in Leon’s comments:

*My wife, she has got all the qualifications. She’s got a certificate three in retail, certificate three in disability, certificate three in aged care. She’s been applying everywhere. They didn’t offer her a job. Until now she’s still applying so she decided to now stay home and let me go and do a diploma in child care. (Leon, Africa, male).*

Overall, participants’ challenges in accessing education affected experiences of integration and health and wellbeing. Barriers to access led to people feeling frustrated and demoralized, particularly in relation to pathways from education to relevant employment opportunities. Experiences of discrimination added to this sense of being locked out of aspirational pathways. However, many participants were actively pursuing aspirational goals via education and were continuing to work hard and overcome these barriers.

### Housing

While in general housing was seen to be affordable in the town, for some participants initial housing provided by settlement services was felt to be inadequate. One person commented on the difference of being housed alone when he had come from a place where he lived in an active community setting. Others, such as Gabi, commented that their families were given houses that were too small or too big compared to the size of the family, and in two instances families were given two separate dwellings when the family was larger than available housing.

*We didn’t have a bedroom. We put our bed between the corridor […]. We came with my mother-in-law. Okay, my mother-in-law’s one bedroom, my son’s in one bedroom. When they want to go to the toilet they have to ask ‘mum, can we pass?’ Yeah, it was not good. (Gabi, Africa, female).*

As settlement continued and people began to move on from their initial housing, generally participants expressed that they found their accommodation satisfactory. Some had issues with the cost of housing and others discussed challenges with finding suitable housing.

*It’s very difficult [to get a house to rent] […] it’s because someone wants those who are employed […] they will ask you ‘do you have pay slips?’ … .. They can’t call you back to talk to you ‘your application was successful’ or maybe ‘your application was not successful’ so now you’ll keep on waiting and waiting and waiting until you get tired, no response at all they will give you. (Pascal, Africa, male).*

Some participants had managed to purchase their own homes and were pleased with that opportunity. This was notably the case for people with Southeast Asian heritage, who had generally been living in The Town for longer periods than those from Africa, and it was reported by one participant that over ten Southeast Asian background families had purchased their own home. One participant also mentioned that people with heritage from Southeast Asia had a good reputation with real-estate agents which meant that they could find appropriate rentals when necessary. This was contrasted with the experiences of the African heritage participants who mentioned difficulty accessing suitable rental accommodation, particularly for those with larger families that could not be easily accommodated in the available housing stock. Furthermore, as Sydney highlights, African participants experienced instances of racism regarding renting homes or trying to borrow money from the bank to buy a house.

*Every time they put in papers, it’s like every African is not clean […] When they do some research, like a documentary, they can’t take anyone who is successful African or a person which is clean and a person which is educated, they usually go to people who -- maybe they are suffering […*], *so any white person or any Australian who does not have to see that, like African can take good care of their house, so go to the house every time it was ‘your application was not successful. Your application is not successful’. (Sydney, Africa, male).*

Experiences of inappropriate housing upon arrival were somewhat detrimental to initial experiences of welcome for participants. These initial barriers continued to prevent positive integration via a sense of inclusion and belonging for some participants due to ongoing discrimination regarding renting a home. However, most participants were able to seek and secure appropriate housing over time, particularly those who were able to successfully navigate systems to purchase a house and feel secure and settled in the town. Discrimination in housing was also reduced over time as the broader population in The Town utilized opportunities to get to know individuals and develop a positive perception of cultural communities.

### Access to services and resources

In this section we consider access to a broad range of services including health services, settlement support services and general services such as transport.

#### Health services

Access to health care is an important SDH. Participants expressed a mixture of satisfaction at having health care available as well as identifying a number of barriers and challenges to access. These challenges included language barriers, difficulties understanding the system, long waiting times for appointments and having to travel to the city for specialist treatment. Understanding the complexity of the health care system can be a challenge for those who are unfamiliar with their rights and access pathways. Yuzana Win described what happened when she was unaware of the nuances of the system in terms of co-payments in visiting her GP when her provider changed:

*Her family doctor is now finished his job and normally when they went to see him they didn’t need to pay the fees … and now a new doctor, they have to pay for it. (Yuzana Win (*via *interpreter), Southeast Asia, female).*

People with specific chronic or acute health issues mentioned that travel to the closest city, Adelaide, was necessary. For some, the nature of their need was immediate and provided for. For others, such as Sydney, the waiting time to be offered an appointment was troubling.

*Like here in [the] Hospital [they say] […] we need to go to Adelaide they put you to waiting list. For example, I have a problem of my teeth so they say ‘okay, so we’ll put you to waiting list’ since … [over a year] … they have not yet called me because they need to do some operation. (Sydney, Africa, male).*

The travel distance to Adelaide was also a challenge for participants who did not have access to a car or to a place to stay in Adelaide. For example, when Emmanuel needed to go to Adelaide for a family health emergency, he had not been there before, but fortunately a fellow member of the Catholic Church volunteered to drive him there.

Language barriers were discussed in relation to accessing health care. Myint Htet Yaza spoke about the issue of finding interpreters who speak the same regional dialect as the patient.

*The only issue is the interpreter sometimes, you know, because they are from a different area and the interpreter on the phone is from a different area where they speak -- it’s the same language but it’s different pronunciation and sometimes could be mistake, like -- and then sometimes the doctor can give them out different medication that will affect them. (Myint Htet Yaza (*via *interpreter), Southeast Asia, male).*

Two female participants described ongoing health complaints that continued to go undiagnosed. Both of these women spoke with the researchers via interpreters indicating possible ongoing language-related systemic barriers to support for their health.

#### Settlement support services

Settlement services provide initial support for people as they arrive and settle in Australia. In rural contexts the service centres are quite small, in some cases run by only one key staff member. The services discussed by participants in this study were quite limited, with some people only having a brief tour of The Town and minimal follow up support. The initial tour and connections to vital elements of settlement such as housing and health care were described as very helpful by most participants. However, in a number of cases participants reported being given only a brief set of supports upon arrival, leading to difficulties accessing key supports such as food, shelter and health care. For example:

*… and then they show us the house, they show us whatever, and they ask us to come the next day at the office, which was a public holiday. After that they ring us and we went to offices, to like [a service provider] and to the bank and show us the hospital, different services where we should go next. I think that’s all. It [wasn’t] a really good explanation for new people to be here because I remember I was lost going [to the] clinic. (Tatiana, Africa, female).*

A majority of participants identified the settlement services as their key place to go for advice or help, particularly during the initial phase of settlement. This point of connection was crucial to successful integration in terms of enabling people to access what they needed for a good life in The Town. Participants who discussed positive connections to settlement services mentioned that they would visit the service to ask for the help they wanted. In many cases participants sought help elsewhere in their cultural and faith communities which showed individuals’ capacity to seek help and make connections. However, it seemed that settlement services could make more use of these connections. In some cases, participants mentioned barriers to connection with communities in The Town due to settlement services failing to advise cultural or faith communities that there would be new people arriving. Access to housing, services, resources and community connections were all related to the ways in which settlement services were provided. However, individuals drew on their personal connections over time to access key elements for good health such as food and housing, as well as finding ways to access appropriate health care. These connections are a strength that settlement services could connect with to strengthen positive integration and health outcomes.

#### Other support services

In terms of other services, The Town has public transport, but this is limited and distances can be quite vast when people need to travel to a neighbouring town or to the city. This was particularly challenging for people who were new to the country and had little option but to walk or catch public transport. Transport was a key issue for many participants in relation to accessing services, education and employment. Some people were limited in their opportunity to attend education or employment because of the distances and lack of either a driver’s licence or car (and associated ongoing costs). For example, Maung U Khaing Thawda had been working in a factory more than an hour’s drive from The Town but had stopped after his transport with other community members was no longer available:

*He was having two friends work together and two of his friends, one has moved to Perth and one has stopped working so he doesn’t have car to go with everybody else so he just stop it too. (Maung U Khaing Thawda (*via *interpreter), Southeast Asia, male).*

In addition to the limitations of distance to access services, specific resources that were culturally important such as food, cosmetics and clothes were also hard to access, particularly for the newer African community:

*It’s very hard to find African food. If you want to afford African food you have to move from [The Town] to Adelaide or to Melbourne in order to get it. It’s very, very hard. (Gasore (*via *interpreter), Africa, male).*

### Secondary migration

The settlement challenges experienced by participants led some participants to consider moving away. For example, Zeyar Htet spoke about community members who were moving to a nearby city for education and employment opportunities.

*There’s a few people that are moving out …*. [*to the city] …*. F*irst things is a job and also there is a few young people that they want to get a high qualification, that they think they can get high qualification if they go to [nearby cities] so, yeah … .also get work as well, like in a big factory and stuff that is close to their house and stuff.* (Zeyar Htet, Southeast Asia, male).

Similar limitations around employment and education were noted by members of the African community as contributing to secondary migration away from the Town.

*I like [the Town] but when my children is going in university, because university we have here is -- it doesn’t have all courses; it has a little bit. When my children is going -- someone, he say he’s going to do dentist and dentist is not there. I think I will be moving. I will going in [nearby cities]* (Paul, Africa, male).

*There is no job. We have now to move. If we stay like this at the end you can find yourself ten years in [the Town] doing nothing’. (Gabi, Africa, female).*

## Discussion

Rural settlement as a policy priority has the potential to support positive outcomes for people from refugee backgrounds. The study revealed that challenges to integration and SDH (as well as successful experiences) were important post-migration health influences for participants and that these factors are key public health issues to be considered in rural settings for refugee resettlement. While a sense of safety and some elements of social connectedness and support were key health facilitators, difficulties in securing employment, mismatched education provision, limitations of service access, and experiences of discrimination are key matters to address in any widening of this approach to resettlement.

Safety is a key social determinant of health [65–67] and also an important element of integration for migrants settling in a new country, most particularly for those with refugee backgrounds many of whom have experienced torture and trauma and long periods of dislocation before arrival [9, 19]. Overall, The Town was considered a good place to raise children and offered a strong sense of safety for participants, which promoted health and wellbeing. Likewise, social connections both within the respective communities and also with other communities in The Town, particularly through religious connections, offered important practical and emotional support and were protective of health and important in building a new life, mirroring a broad literature on the importance of strong social ties for health and wellbeing [68–75].

However, there were barriers to building these ties such as language difficulties and cultural differences. Racism impacted on people’s lives, particularly those from Africa, undermining a sense of belonging, safety, and in turn effective integration [76, 77]. There is a substantial literature highlighting the detrimental impact of racism on health in general [78–82] as well as for refugees specifically [57, 83–86], including in rural resettlement [41, 76, 77, 87]. Community development initiatives such as community arts projects and anti-racism curriculum in schools are important in these contexts. Settlement services have an important role to play in systematically developing links and relationships and by connecting newcomers to a broad range of communities. Community organizations are also enormously important in helping new arrivals to adapt and flourish in Australia and also act as bridges between communities and need to be adequately resourced to do so.

Unemployment and underemployment are key issues more broadly in rural towns in Australia and internationally and have also been identified as particular issues for regional refugee resettlement nationally [9, 41, 58, 87–90] and internationally [11]. Difficulties in accessing secure long-term employment affected resettlement and integration experiences of the participants in this study. Employment levels were generally not commensurate with the education levels of the participants, a trend which has been recognised for some time [43]. Participants, including those in the Town for some years, found employment challenging to secure, particularly in relation to not having qualifications recognised and being hired mostly for menial and manual labour rather than more highly paid technical or professional roles. Although people’s willingness to work in less desirable roles may be helpful for the region and businesses concerned, this needs to be balanced with opportunities for people from refugee backgrounds to realize their employment aspirations and potential [57]. Unemployment and insecure or poor working conditions are well known SDH [91–93]. For the research participants a lack of access to secure work was frustrating, disheartening and stressful and also meant limited financial resources for the other ‘tasks’ of resettlement, as well as challenges to identity and meaning. Difficulties securing employment was also identified as a potential motivation to leave the Town. There are considerable social and economic benefits of successful resettlement for both new arrivals and receiving communities. As such, successfully supporting new arrivals into employment is an important way to retain skills in the Town and the other contributions that new arrivals make.

Volunteering was shown to be a pathway to employment in this study. Volunteering can also be a mechanism to increase connectedness, and a sense of belonging and self-fulfilment [94]. Our findings also suggest that there is potential for people with refugee backgrounds to be taken advantage of. The key difference between paid and volunteer work is the financial aspect which flows on to other SDH such as housing, access to services, access to education, and support with travel costs (which is vital in rural Australia). Given that one of the major rationales for regional resettlement is to fill needed employment gaps [58], matching migrant’s skills to employment opportunities in an area is an important key for success. Focused policy and government services could support the role of employers, services providers and agencies to include support of systematic volunteering pathways to paid employment and protections from exploitation [88, 95], alongside systematic strategies to build social networks important for securing work.

The failure to recognise prior education by Australian systems results in people being excluded from employment opportunities commensurate with their capacity, and feeds into the employment niche of low-pay, menial work for people from refugee backgrounds in Australia [96]. Participants in our study reported frustration at not having prior qualifications recognised and streamlined processes for qualification recognition is crucial. While some participants were already highly educated, another cohort of participants described less opportunity for education in their previous home country, and they also found the English language classes in The Town to be insufficient to their needs. For those who pursued education after difficulties in securing work only to still find difficulties finding work, the education-employment churn was very disheartening. Greater flexibility in English language classes to cater for new arrivals and for settlement services to work in partnership with providers to facilitate this may be useful, as would English classes connecting with established cultural or religious communities and employers to build community networks and strengths.

Housing is a SDH for refugees [97] and previous studies have highlighted challenges for refugees in securing appropriate housing [53, 98–101]. There were mixed experiences of housing for participants. Initial housing provided by the government through the settlement services was sometimes inadequate or a mismatch for individuals or families. Over time, most participants were able to secure suitable rental housing, although some of the African background participants mentioned difficulties with discrimination relating to rental options. There was a positive reported outcome for the Southeast Asian background community who had lived in the town for a longer time - around 10 families had purchased their own house. The community and family support discussed in the Southeast Asian background community was one reason they were able to work towards and achieve the goal of home ownership.

Access to health care and other services can be particularly challenging in rural or remote areas for refugees [49, 102, 103], and this was the case for participants in this study. These challenges were amplified by the multiple barriers to access that people from refugee backgrounds experience in resettlement [104]. The language barriers to accessing health and other services and difficulties securing interpreters in specific dialects suggests the need for accessible routes to engaging appropriate interpreters for people who would like this support. Beyond health care, participants also found accessing specific resources and services including culturally appropriate food and clothing to be challenging in the rural setting. Some participants described their solutions to this by using their community connections to join together to source items from the city, or to share travel arrangements for travel to the city or to other towns for work. Participants who had been living in the town for longer periods of time were more likely to have formed arrangements to navigate these challenges, reinforcing that initial settlement provisions could perhaps aim to support these needs and the importance of social networks for rural residents.

Secondary migration is fairly common during the early years of settlement for people from refugee backgrounds [90, 105, 106]. People may choose to relocate particularly for access to employment or education [107]. In this study, those who moved *to* The Town did so to link with communities already living there, indicating that community support is a strength to build on, and that indeed community and family connection is vital [106]. Most participants in this study had arrived straight to the rural town via the rural resettlement initiative and many mentioned their friends or family who had moved away already, and the potential moves that they themselves might consider – largely in response to difficulties securing employment. Secondary migration *away* from rural areas not only defeats the initial purpose of policy priorities for rural resettlement, but it also impacts on the individuals and families involved [108]; the impact of struggling to find work or to access education, followed by the challenges of uprooting one’s-self and family after prior relocation via a refugee migration should not be underestimated.

In many cases, the term integration is assumed to mean that a newcomer should adapt to and adopt the social norms of the place they settle in [109], however integration is a two-way process where receiving communities also adapt and the ways that integration can occur are bound by the ways in which structures of power are embedded in social practices and institutions [15, 110]. These systems of power affect equitable access in many ways, particularly for people from refugee backgrounds in resettlement. As an example, in Australia the legal processes that frame resettlement affect which services are available, in what form and for whom. In our study we found significant structural barriers to integration and hence SDH were affected. These included barriers to recognition of qualifications, to securing meaningful and ongoing employment, and to accessing key necessities of life such as health services and housing. In this way a SDH approach, as reflected in Dahlgren and Whitehead’s [14] model and further examined in the WHO Commission on the Social Determinants of Health [111], is particularly useful in highlighting the importance of considering receiving community factors (such as levels of racial intolerance or availability of services), and the distribution of power and resources and the impact of general socioeconomic, cultural and environmental conditions on the health of individuals from refugee backgrounds [5, 29, 30].

### Strengths and limitations

The study involved participants from a number of cultural groups settled in the same town for a range of time periods. This enabled an exploration of variations of experiences within the same town and focused on the lived experience of refugees themselves. The sampling approach and the use of qualitative method meant that research participants included those who were not currently engaged with service organisations resulting in data that was rich and enabled deep understanding of individual resettlement stories. However, interviewing people connected to the known local leaders and the snowballing approach (which relies on social networks and is therefore not a random sampling approach) may still have missed some less connected people. Likewise, the scope of our research did not extend to views of the broader regional community, which would have given further insight into the two-way aspect of integration. Previous studies have highlighted the impact of receiving community views on the experiences of new arrivals e.g. [15, 41, 76, 77, 89, 112] and, for example, the reports from participants in this study of racial discrimination further indicates the importance of examining this. As such this would be an area of fruitful future research.

Our study included participants from particular cultural backgrounds in a specific rural town. Their experiences may be similar to those with similar backgrounds and current living arrangements; however, local contextual detail is relevant. As such the findings may not be transferrable to people in other rural areas or with different cultural or experiential backgrounds.

## Conclusions

Conceptually, integration and SDH are directly linked through factors such as employment, education, housing, and social connections, and this study provided further empirical evidence of these links. The findings suggest that SDH, and therefore key elements of integration, may be challenged in regional settings for people from refugee backgrounds, partially as a result of settlement policies. The links between SDH and integration are important factors to be considered by policy makers focused on successful regional resettlement initiatives. The possible positive outcomes for newcomers and receiving communities are wide-ranging, but these need to be supported by clear policy directives that address current systemic barriers to successful integration, as well as reflecting the local contexts of resettlement.

## Supplementary Information

**Additional file 1.** Supplementary file – Interview guide. Rural settlement experiences of people from refugee backgrounds: Semi-structured interview guide.

## Data Availability

Due to ethical concerns, supporting data cannot be made openly available. Please contact the author for further information about the data and conditions for access.

## Abbreviation

SDH: Social determinants of health
