# Supplementary material for: Integration and social determinants of health and wellbeing for people from refugee backgrounds resettled in a rural town in South Australia: a qualitative study
Source: BMC Public Health. 2020 Nov 13;20:1700. doi: 10.1186/s12889-020-09724-z (PMC7663864; doi:10.1186/s12889-020-09724-z)
Supplement: Supplementary file 1 — Additional file 1. Supplementary file – Interview guide. Rural settlement experiences of people from refugee backgrounds: Semi-structured interview guide. [file 12889_2020_9724_MOESM1_ESM.docx]

**Rural settlement experiences of people from refugee backgrounds:**

**Semi-structured interview guide**

*General demographic background*

- gender, cultural background, education, employment, family/marital status

*Arrival to Australia*

- When did you arrive in Australia?
- where was your initial place of arrival?
- after your initial settlement, did you move to other places (within SA or interstate), why (including existing links to area)?
- What have been your experiences of the settlement processes in the area?
  - what have you liked about living in the area?
  - any issues experienced living in the area?
- [if not already raised] What have been your experiences with:
- housing
- employment
- education
- service access/settlement support
- social networks and engagement (including specific ethnic community and receiving community)
- safety
- health
- other
- Ongoing experiences

*Impact on life, future migration intentions*

- What were the impacts of settlement experiences on:
- health and wellbeing
- relationships and sense of belonging
- other – e.g. education, employment, housing
- onward migration
- How have you managed these?
- Anything else about your experiences of living in a regional setting?
